# High Expression of FLOT1 Is Associated with Progression and Poor Prognosis in Hepatocellular Carcinoma

**DOI:** 10.1371/journal.pone.0064709

**Published:** 2013-06-26

**Authors:** Shi-Hong Zhang, Chan-Juan Wang, Ling Shi, Xing-Hua Li, Jing Zhou, Li-Bing Song, Wen-Ting Liao

**Affiliations:** 1 Department of Pathology, Nanfang Hospital, Southern Medical University, Guangzhou, Guangdong, China; 2 State Key Laboratory of Oncology in Southern China, Department of Experimental Research, Sun Yat-sen University Cancer Center, Guangzhou, Guangdong, China; 3 Department of Laboratry Medicine, the First Affiliated Hospital of Sun Yat-sen University, Guangzhou, Guangdong, China; 4 Department of Pathology, School of Basic Medical Sciences, Southern Medical University, Guangzhou, Guangdong, China; University of Navarra School of Medicine and Center for Applied Medical Research (CIMA), Spain

## Abstract

**Background:**

The flotillin family member flotillin-1 (FLOT1) encodes a caveolae-associated, integral membrane protein that belongs to lipid raft family and involves in vesicular trafficking and signal transduction. However, the role of FLOT1 in development and progression of cancer remains largely unknown. The present study was aimed to investigate the clinical and prognostic significance of FLOT1 in hepatocellular carcinoma (HCC).

**Methods:**

Real-time PCR and western blot analyses were applied to examine FLOT1 expression in fourteen HCC cell lines and one normal hepatic cell line, ten pairs of primary HCC and matched adjacent noncancerous liver tissues from the same patient. Immunohistochemistry (IHC) was performed to examine FLOT1 protein expression in paraffin-embedded tissues from 196 HCC patients. Statistical analyses were applied to evaluate the diagnostic value and associations of FLOT1 expression with clinical parameters.

**Results:**

FLOT1 expression was evidently up-regulated in HCC tissues compared with that in the matched adjacent noncancerous liver tissues. In the 196 cases of tested HCC samples, FLOT1 protein level was positively correlated with Tumor size (*P* = 0.025), clinical stage (*P*<0.002), CLIP stage (*P*<0.001), vascular invasion (*P*<0.001), relapse (*P*<0.001), and serum AFP levels (*P* = 0.025). Patients with higher FLOT1 expression had shorter overall survival time, whereas those with lower FLOT1 expression had longer survival time.

**Conclusions:**

Our study demonstrated FLOT1 is associated with aggressive characteristics of HCC, and suggested the possibility of its use as a prognostic marker in patients with HCC.

## Introduction

Hepatocellular carcinoma (HCC) is the fifth most common cancer in the world and represents the third leading cause of cancer mortality worldwide, with a only 30% to 40% five-year postoperative survival rate [1], [2]. Most of the cases (85%) occur in developing countries, with the highest incidence rates Southeast Asia and sub-Saharan Africa [3]. Major risk factors for HCC include both environmental factors (such as infection with HBV and alcoholic liver disease) and genetic/epigenetic alterations [1], [2], [4]. However, the molecular mechanism of its development and progression remains largely unknown. Thus, it is critical to understand the etiology and to illustrate the mechanisms underlying HCC initiation and progression, and further identify valuable diagnostic and prognostic markers as well as novel therapeutic strategies.

Lipid rafts function as physical platforms for various molecules that involved in a variety of biologic processes by serving as organizing centers for the assembly of signaling molecules into functional complexes [5]. Due to its essential function in a variety of biological processes, the protein markers of lipid raft has been well documented to involved in initiation and progression of human cancers [6], [7], [8]. The flotillin protein family members, including Reggie-1/FLOT2 and reggie-2/FLOT1, are essential markers of lipid rafts [9], [10], [11]. These proteins are ubiquitously expressed and play important roles in a wide variety of cellular processes such as membrane receptor signaling, membrane trafficking, actin cytoskeleton reorganizations, cell adhesion and cell motility [5], [12]. For example, flotillin proteins were involved in activation of insulin signaling and epidermal growth factor receptor signaling through recruitment of receptor kinases to lipid rafts [13], [14]. Besides their functions in the cellular and organelle membranes, flotillin proteins also participated in development and progression of human cancer [15], [16]. Recently, deregulation of FLOT1 was found in epithelium-originated cancer, including breast cancer, colorectal cancer, as well as esophageal squamous cell carcinoma [16], [17], [18]. Ectopic expression of FLOT1 promoted proliferation of esophageal squamous cell carcinoma cell lines, whereas silencing FLOT1 inhibited the proliferation and tumorigenicity of breast cancer cells both in vitro and in vivo [16], [17]. These findings suggest that FLOT1 plays a dominant positive role in the development and progression of epithelium-originated cancers. However, whether FLOT1 deregulation also occurs in human HCC remains unclear. To address this question, we sought to investigate the expression of FLOT1 in HCC and evaluated its clinicopathologic and prognostic significance in 196 archived HCC samples.

## Materials and Methods

### Ethics Statement

For the use of clinical materials for research purposes, prior patients' consents and approval were obtained from the Sun Yat-sen University and Cancer Center Institutional Board. All samples were collected and analyzed with prior written informed consents from the patients. Paraffin-embedded, archived HCC samples were obtained from 196 patients who were histopathologically and clinically diagnosed at the Sun Yat-sen University Cancer Center between January 2004 and December 2006. The median age of the patients was 48 years (range, 22–78 years old). The median follow-up time was 34.8 months (range, 2–115 months). Ten pairs of HCC biopsies with matched adjacent non-cancerous normal liver tissues were frozen and stored in liquid nitrogen until further use. The disease stages of all the patients were classified or reclassified according to the American Joint Committee on Cancer (AJCC) TNM staging system [19].

### Cell culture

Fourteen HCC cell lines (Huh7, M3, M6, QGY7703, PLC, HCCC9810, 97L,97H, QGY7701, QGY7402, Bel7404, QGY7721,HepG2, and Hep3B) and one normal hepatic cell line (Lo2) were purchased from the ATCC Cell Biology Collection and were maintained in Department of Experimental Research, Sun Yat-sen University Cancer Center. Cells were cultured in Dulbecco's modified Eagle medium (DMEM, Invitrogen, Carlsbad, CA) supplemented with 10% fetal bovine serum (FBS, HyClone, Logan, UT) and 1% penicillinestreptomycin (Invitrogen, Grand Island, NY) at 37°C with 5% CO2.

### RNA extraction and Real-time polymerase chain reaction (PCR)

Total RNA from tissue samples were extracted using the Trizol reagent (Invitrogen, Carlsbad, CA) according to the manufacturer's instruction. The extracted RNA was pretreated with RNase-free DNase, and 2 Ag RNA from each sample was used for cDNA synthesis primed with random hexamers. For PCR-mediated amplification of FLOT1 cDNA, an initial amplification using FLOT1-specific primers was done with a denaturation step at 95°C for 10 min followed by 30 denaturation cycles at 95°C for 60 s, primer annealing at 55°C for 30 s, and primer extension at 72°C for 30 s. On completion of the cycling steps, a final extension at 72°C for 5 min was carried out before the reaction was stopped and stored at 4°C. Real-time PCR was then employed to determine the fold increase of FLOT1 mRNA in each of the primary HCC tumors relative to the paired noncancerous liver tissues, with each pair taken from the same patient. Reverse transcription-PCR and real-time PCR primers were designed using the Primer Express. Sequences of the real-time PCR primers were: FLOT1, forward: 5′-CCCATCTCAGTCACTGGCATT-3′ and reverse: 5′-CCGCCAACAT CTCCTTGTTC-3′ [16]. Expression data were normalized to the geometric mean of the housekeeping gene *GAPDH* [20] and calculated as 2^−[(Ct of *FLOT-1*)−(Ct of *GAPDH*)]^, where C_t_ represents the threshold cycle for each transcript.

### Western blot

Western blots were performed according to standard methods as described previously [21]. Briefly, equal amounts of protein were separated by electrophoresis on a 10.5% sodium dodecyl sulfate polyacrylamide gel and electrotransferred from the gel to a nitrocellulose membrane. After blocking with 5% milk solution in Tris-buffered saline with Tween (TBST) for 1 hour, the membrane was incubated with primary antibody against using anti-FLOT1 polyclonal antibody (1∶500, Sigma, Saint Louis, MI) for 2 hours at room temperature. Anti-α-tubulin mouse monoclonal antibody (dilution, 1∶1000; Santa Cruz Biotechnology, Santa Cruz, Calif) was used as an internal loading control. After washing with TBS-T, the membrane was incubated with secondary antibody against rabbit immunoglobulin G or mouse immunoglobulin G; then, it was examined with the enhanced chemiluminescence detection system (Amersham Biosciences Europe, Freiberg, Germany) according to the manufacturer's instructions.

### Immunohistochemical (IHC) analysis

IHC analysis was carried out similarly to previously described methods [21]. Briefly, tissue sections were incubated with anti-FLOT1 antibody (1∶500; Sigma, Saint Louis, MI) overnight at 4°C. For negative controls, the anti-FLOT1 antibody was replaced with normal non-immune serum. The sections were reviewed and scored independently by two observers, based on both the proportion of positively stained tumor cells and the intensity of staining [21]. The proportion of tumor cells was scored as follows: 0 (no positive tumor cells), 1 (<10% positive tumor cells), 2 (10–50% positive tumor cells) and 3 (>50% positive tumor cells). The intensity of staining was graded according to the following criteria: 0 (no staining); 1 (weak staining = light yellow), 2 (moderate staining = yellow brown) and 3 (strong staining = brown). The staining index (SI) was calculated as staining intensity score×proportion of positive tumor cells. Using this method of assessment, we evaluated the expression of FLOT1 in benign liver tissues and HCC lesions by determining the SI, which scores as 0, 1, 2, 3, 4, 6 and 9. Cutoff values for FLOT1 were chosen on the basis of a measure of heterogeneity with the log-rank test statistical analysis with respect to overall survival. An optimal cutoff value was identified: the SI score of ≥4 was used to define tumors as having high FLOT1 expression and ≤3 as having low expression of FLOT1. To account for the inconsistencies in IHC stain intensities, the mean optical density (MOD) method, which was used for the scoring of the staining intensity, was applied in the current study. In brief, the stained slides were evaluated at 200× magnification using the SAMBA 4000 computerized image analysis system with Immuno 4.0 quantitative program (Image Products International, Chantilly, VA). Ten representative staining fields of each tumor sample were analyzed to determine the MOD, which represented the concentration of the stain or proportion of positive pixels within the whole tissue. A negative control for each staining batch was used for background subtraction in the quantitative analysis. The data were statistically analyzed using t-test to determine the differences in average MOD values between different groups of tissues. *P*<0.05 was considered significant.

### Statistical analyses

All statistical analyses were performed using the SPSS 11.0 statistical software package. Comparisons between groups for statistical significance were performed with a two-tailed paired Student's t test. The chi-square test was used to analyze the relationship between FLOT1 expression and clinicopathologic features. Bivariate correlations between variables were calculated by Spearman's correlation coefficients. Survival curves were plotted by the Kaplan-Meier method and compared using the log-rank test. Survival data were evaluated using univariate and multivariate Cox regression analyses. *P*<0.05 in all cases was considered statistically significant.

## Results

### FLOT1 is up-regulated in HCC

**Figure 1:**
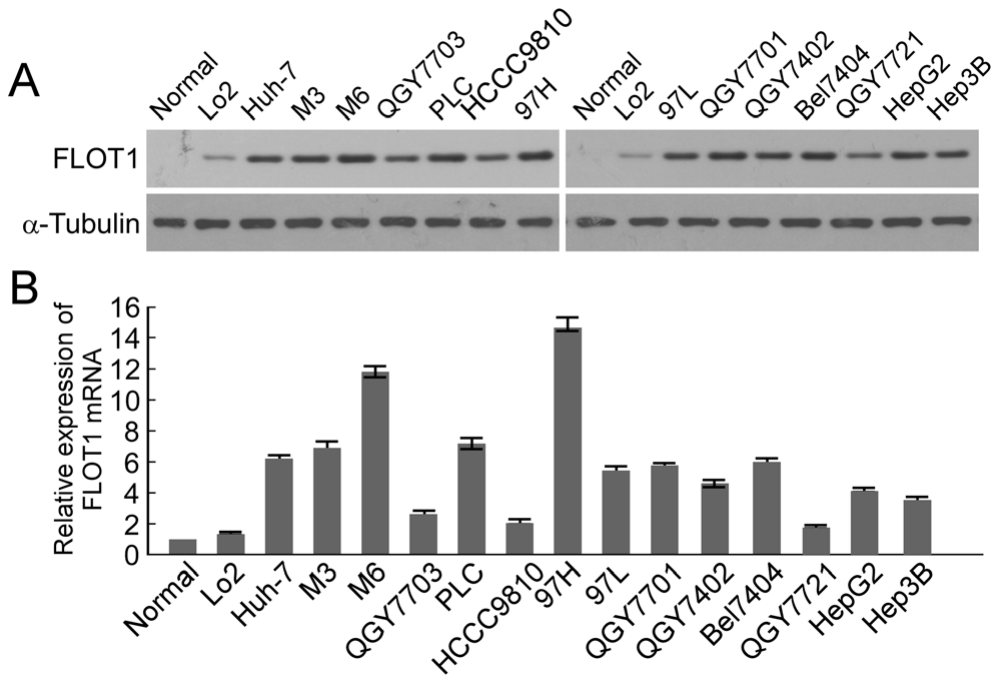
Expression analysis of FLOT1 in HCC cell lines and normal hepatic cell line. (**A**) Expression of FLOT1 protein in Lo2, cultured HCC cell lines (Huh7, M3, M6, QGY7703, PLC, HCCC9810, 97L,97H, QGY7701, QGY7402, Bel7404, QGY7721,HepG2, and Hep3B), and one case of normal liver tissue by Western blotting. (**B**) Expression of FLOT1 mRNA in the Lo2, normal liver tissue, and cultured HCC cell lines real-time reverse transcription-PCR. Expression levels were normalized for GAPDH. Columns, mean from three parallel experiments; bars, SD.

Western blotting analysis revealed an evidently higher level of FLOT1 expression in all fourteen HCC cell lines than in normal liver cell line Lo2 and one case of normal liver tissue, which was used for purposes of comparison (Figure 1A). To clarify whether FLOT1 up-regulation was occurring at transcriptional level, additional Real-time RT-PCR analyses were performed. Figure 1B demonstrated that the mRNA level of FLOT1 in all HCC cell lines was obviously higher than that in normal liver tissue and cell line. Additionally, we noted that FLOT1 expression was relatively higher in two highly metastatic HCC cell lines (MHCC97H and HCCLM6) than that in other HCC cell lines (Figure 1A and 1B). These results demonstrated that FLOT1 expression was elevated at both the mRNA level and the protein levels in the HCC cancer cell lines.

**Figure 2:**
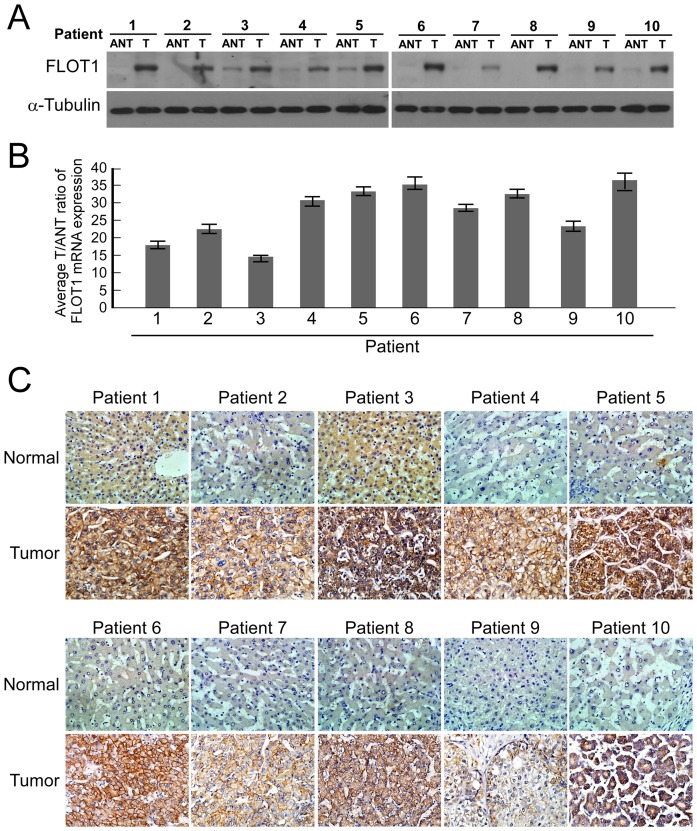
Upregulation of FLOT1 in HCC tissues. (**A and B**) Expression of FLOT1 protein and mRNA in ten paires of HCC tissues (T) and matched adjacent non-cancerous tissues (N) in the same patient determined by Western blotting (A) and real-time PCR (B), respectively. (**C**) Expression of FLOT1 protein in each of the primary SGC tissues and adjacent non-cancerous tissues by immunohistochemistry.

In order to determine whether the up-regulation of FLOT1 in HCC cell lines is clinically correlated with HCC progression, we did Western blotting analysis on ten pairs of matched normal liver tissue and HCC samples. As shown in Figure 2A, FLOT1 was found to be differentially overexpressed in all ten examined human primary HCC samples paired with normal liver tissues from the same patients. By real-time RT-PCR analysis, the tumor/adjacent non-cancerous (T/N) ratio of FLOT1 mRNA expression was >2-fold in all these samples, and the highest ratio was up to about 40-fold (Figure 2B). These findings are consistent with the results obtained in our immunohistochemical analysis (Figure 2C).

### Overexpression of FLOT1 protein in archived HCC samples

**Figure 3:**
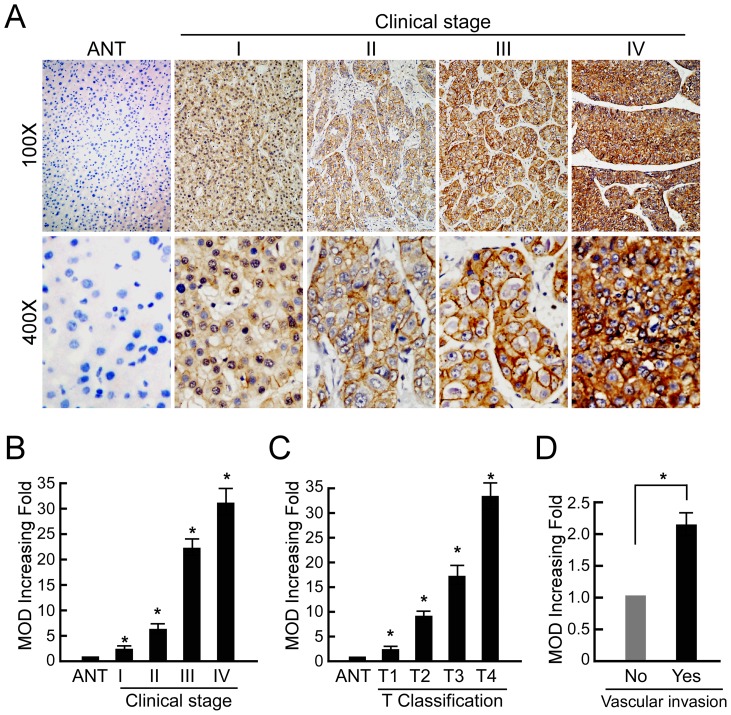
FLOT1 protein overexpression in archived paraffin-embedded SGC tissue sections by immunohistochemistry. (**A**) Representative images from immunohistochemistry analyses of FLOT1 expression in normal liver tissue and different clinical stages of HCC tissues. (**B**) Statistical analyses of the average MOD of FLOT1 staining between normal liver tissues and HCC specimens of different clinical stages. (**C**) Statistical analyses of the average MOD of FLOT1 staining between normal liver tissues and HCC specimens of different T classification. (**D**) Statistical analyses of the average MOD of FLOT1 staining between non-vascular invasion group and vascular invasion group. * *P*<0.05.

To determine the role of FLOT1 in the clinical progression of HCC, IHC analysis was performed in 196 paraffin-embedded, archived HCC tissue samples, including 18 cases of stage I, 73 cases of stage II, 102 cases of stage III, and 3 cases of stage IV tumors. As shown in Figure 3A, FLOT1 protein was detected in 185 of 196 (94.4%) cases. High levels of FLOT1 were present in cancerous lesions in the primary HCC tumors. In contrast, FLOT1 was negatively or only weakly detectable in normal liver tissues. Quantitative IHC analysis revealed that the MOD values of FLOT1 staining in all primary SGC were higher than that in control normal tissues. In addition, the MOD values of FLOT1 staining was significantly increased along with the progression of tumor grades I to IV (*P*<0.001, Figure 3B) and T classification 1 to 4 (*P*<0.001, Figure 3C). Moreover, MOD values of FLOT1 staining was obviously higher in vascular invasion group than that in non-vascular invasion group (*P*<0.01, Figure 3D)

### Increased FLOT1 expression correlates with clinicopathologic features of HCC

We further examined the possible correlations between expression levels of FLOT1 and clinical features of HCC. As summarized in Table 1, analyzing of 196 primary HCC samples indicated that FLOT1 expression was strongly correlated with tumor size (*P* = 0.025), clinical stage (*P*<0.002), CLIP stage (*P*<0.001), vascular invasion (*P*<0.001), relapse (*P*<0.001), and serum AFP levels (*P* = 0.025). Spearman correlation analysis (Table 2) convinced that high FLOT1 expression level was closely correlated with larger tumor size (R = 0.160, *P* = 0.013), advanced clinical stage (R = 0.234, *P*<0.001), CLIP stage (R = 0.329, *P* = 0.002), vascular invasion (R = 0.32, *P*<0.001), relapse (R = 0.525, *P*<0.001), and serum AFP levels (R = 0.160, *P* = 0.013). However, our analyses did not show significant associations between FLOT1 expression and other clinical features including age, gender, hepatitis history, liver cirrhosis and tumor multiplicity (Table 2).

**Table 1:** Correlations between FLOT1 expression and clinicopathologic characteristics of hepatocellular carcinoma patients.

Characteristics	No. cases	FLOT1 expression		Chi-square *p*-value
		Low or none (%)	High (%)	
**Gender**				0.090
**Male**	176	88(50.0)	88(50.0)	
**Female**	20	6(30.0)	14(70.0)	
**Age(years)**				0.314
**≤48**	99	51(51.5)	48(48.5)	
**>48**	97	43(44.3)	54(55.7)	
**Hepatitis histrory**				0.971
**Yes**	167	80(47.9)	87(52.1)	
**No**	29	14(48.3)	15(51.7)	
**Liver cirrhosis**				0.146
**Yes**	145	74(51.0)	71(49.0)	
**No**	51	20(39.2)	31(60.8)	
**Tumor size (cm)**				0.025
**≤5**	86	49(57.0)	37(43.0)	
**>5**	110	45(40.9)	65(59.1)	
**Tumor multiplicity**				0.081
**Single**	143	74(51.7)	69(48.3)	
**Multiple**	53	20(37.7)	33(62.3)	
**Clinical Stage**				0.002
**I**	18	15(83.3)	3(16.7)	
**II**	73	39(53.4)	34(46.6)	
**III**	102	38(37.3)	64(62.7)	
**IV**	3	2(66.7)	1(33.3)	
**CLIP Stage**				<0.001
**0**	44	35(79.5)	9(20.5)	
**1**	45	20(44.4)	25(55.6)	
**2**	33	15(45.5)	18(54.5)	
**3**	29	11(37.9)	18(62.1)	
**4**	24	8(33.3)	16(66.7)	
**5**	18	4(22.2)	14(77.8)	
**6**	3	1(33.3)	2(66.7)	
**Vascular invasion**				<0.001
**Yes**	91	28(30.8)	63(69.2)	
**No**	105	66(62.9)	39(37.1)	
**Relapse**				<0.001
**Yes**	75	11(14.7)	64(85.3)	
**No**	121	83(68.6)	38(31.4)	
**AFP**				0.025
**<400 ug/L**	135	92(30.8)	101(30.8)	
**≥400 ug/L**	61	2(66.7)	1(33.3)	
**Patient survival**				<0.001
**Alive**	52	92(30.8)	101(30.8)	
**Deceased**	144	2(66.7)	1(33.3)	

**Table 2:** Spearman correlation analysis between FLOT1 and clinical pathologic factors.

Characteristics	FLOT1 expression level	
	Correlation coefficient	*p*-value
**Gender**	0.121	0.045
**Age(years)**	0.072	0.158
**Hepatitis histrory**	0.003	0.485
**Liver cirrhosis**	0.104	0.074
**Tumor size (cm)**	0.160	0.013
**Tumor multiplicity**	0.125	0.041
**Clinical stage**	0.234	<0.001
**CLIP**	0.329	0.258
**Vascular invasion**	0.320	<0.001
**Relapse**	0.525	<0.001
**AFP**	0.160	0.013
**Patient survival**	0.395	<0.001

### High FLOT1 expression is associated with poor prognosis of patients with HCC

**Figure 4:**
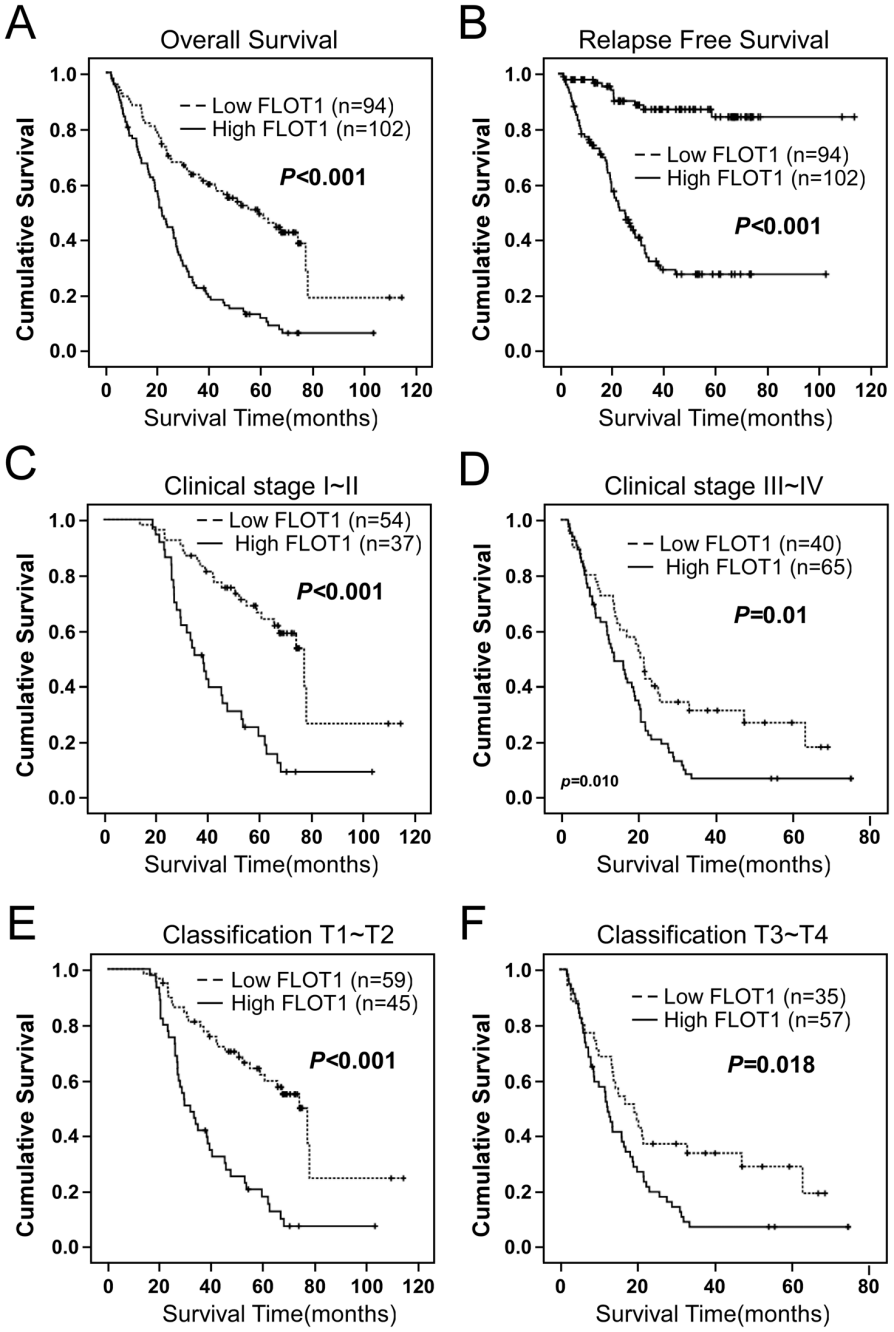
Kaplan-Meier curves with univariate analyses (log-rank). (**A**) The overall survival of patients who had HCC with low FLOT1 expressing tumors (bold lines) versus high FLOT1 expressing tumors (dashed lines). The cumulative 5-year survival rate was 41.5% (95%CI,35.652%∼47.256%) in the low FLOT1 group, whereas it was only 6.7% (95%CI, 3.982%∼9.426%) in the high FLOT1 group. (**B**) The relapse free survival plot of patients with low FLOT1 expressing tumors versus high FLOT1 expressing tumors. The statistical significance of the difference between survival curves for patients with FLOT1 high- and low-expressing tumors was compared between patients with clinical stage I–II disease (**C**) versus stage III–IV disease (**D**) and between patients with tumors that were classified as T1–T2 (E) versus T3/T4 (F). *P* values were calculated by using log-rank tests.

Kaplan-Meier analysis and the log-rank test were used to calculate the effect of FLOT1 expression on survival. The log-rank test showed that the survival time was significantly different between these two groups. Patients with low FLOT1 expression had longer survival times, whereas those with high FLOT1 expression had shorter survival times (Figure 4A, log-rank, *P* = 0.001). The cumulative 5-year survival rate was 41.5% (95%CI,35.652%∼47.256%) in the low FLOT1 group, whereas it was only 6.7% (95%CI, 3.982%∼9.426%) in the high FLOT1 group. Importantly, patients with low FLOT1 expression had a better relapse-free survival (Figure 4B, log-rank, *P* = 0.001). Univariate Cox regression analyses revealed that higher level of FLOT1, Tumor size, Tumor multiplicity, clinical Stage, CLIP stage, vascular invasion, relapse as well as serum AFP levels were all were worse predictors for HCC patients (Table 3). In addition, multivariate Cox regression analysis revealed that tumor multiplicity, clinical stage, CLIP stage, vascular invasion and FLOT1 expression were independent prognostic markers for HCC (Table 4).

**Table 3:** Univariate analysis of different prognostic parameters in patients with hepatocellular carcinoma by Cox-regression analysis.

Characteristics	Univariate analysis	
	*p*-value	HR (95% CI)
**Gender**	0.789	0.927(0.534–1.611)
**Age(years)**	0.788	1.046(0.753–1.453)
**Hepatitis histrory**	0.778	1.069(0.673–1.699)
**Liver cirrhosis**	0.777	1.056(0.728–1.529)
**Tumor size (cm)**	<0.001	2.405(1.695–3.413)
**Tumor multiplicity**	0.010	1.618(1.122–2.333)
**Clinical Stage**	<0.001	2.967(2.259–3.897)
**CLIP**	<0.001	1.634(1.465–1.822)
**Vascular invasion**	<0.001	3.760(2.645–5.345)
**Relapse**	<0.001	5.009(3.435–7.304)
**AFP**	0.007	1.614(1.139–2.288)
**FLOT1 expression**	<0.001	2.759(1.950–3.905)

**Table 4:** Multivariate analysis of different prognostic parameters in patients with hepatocellular carcinoma by Cox-regression analysis.

Characteristics	Multivariate analysis	
	*p*-value	HR (95% CI)
**Gender**	0.143	0.643(0.355–1.162)
**Age(years)**	0.540	0.894(0.626–1.278)
**Hepatitis histrory**	0.483	1.184(0.739–1.897)
**Liver cirrhosis**	0.060	0.677(0.451–1.017)
**Tumor size (cm)**	0.803	1.056(0.688–1.623)
**Tumor multiplicity**	0.025	0.559(0.336–0.930)
**Clinical Stage**	<0.001	2.155(1.494–3.108)
**CLIP**	0.009	1.326(1.072–1.640)
**Vascular invasion**	0.026	1.821(1.075–3.085)
**Relapse**	0.070	1.597(0.962–2.652)
**AFP**	0.787	1.063(0.681–1.661)
**FLOT1 expression**	0.017	1.605(1.089–2.367)

In addition, the prognostic value of FLOT1 expression was analyzed when stratifying the patients according to the clinical stage and T classification Because only 5 samples in subgroups with distant metastasis and 13 cases in subgroups with lymph node involvement, the overall survival was not analyzed stratified by M or N classification. As shown in Figure 4, significantly different outcomes based on FLOT1 expression were compared in patient subgroups with clinical stages I–II (Figure 4C, *P*<0.001) and clinical stages III–IV (Figure 4D, *P* = 0.01). Similar results were obtained for patient subgroups with T1–T2 (Figure 4E, *P*<0.001) and T3–T4 (Figure 4F, *P* = 0.018). Taken together, these results indicate that FLOT1 could be helpful to evaluate the prognosis in HCC patients.

## Discussion

In the present study, we provide the first evidence that elevated expression of FLOT1-1 protein is correlated with poor prognosis of patients with HCC. Our data demonstrated that FLOT1 is up-regulated at both transcriptional and translational levels, in HCC cell lines as compared with normal liver cell line and normal liver tissue. Paired HCC lesions and adjacent noncancerous tissues displayed significantly different expression levels of FLOT1, with the cancer lesions displaying obviously higher expression of FLOT1. Immunohistochemistry staining indicated that the high expression level of FLOT1 protein in histological sections is strongly correlated with aggressive characteristics of human HCC (tumor size, advanced stages, vascular invasion and relapse) and reduced survival time of patients with HCC. Our data implicate that overexpression of FLOT1 protein may be a common feature in HCC and can serve as an independent prognostic marker to identify patients with poor clinical outcome.

FLOT1 encodes a caveolae-associated, integral membrane protein that belongs to lipid raft family and involves in vesicular trafficking and signal transduction [22]. Overexpression of FLOT1 could increase the number of lipid rafts, whereas knockdown of FLOT1 disrupted lipid raft formation [16]. The essential roles of FLOT1 in tumourigenesis have been revealed recently [16], [17], [18], [23]. It has been reported that FLOT1 was a regulator of ErbB2 in breast cancer [24]. In addition, silencing FLOT1 inhibited the proliferation and tumorigenesis of breast cancer cells both in vitro and in vivo, through inhibition of FOXO3a [17]. In contrast, overexpression of FLOT1 increased cell proliferation, anchorage-independent growth, and invasive ability through activation of NF-κB signaling in esophageal squamous cell carcinoma cells [16]. Moreover, in breast cancer and esophageal squamous cell carcinoma, overexpression of FLOT1 could be used as a valuable maker for prediction of poor prognosis of patients [16], [17]. These findings suggested an oncogenic role of FLOT1 in human cancers. In the present study, FLOT1 was found to be upregulated both in HCC cell lines, especially those with highly metastatic potential, and tissue samples as compared with that in normal cell lines and normal liver tissues. Importantly, high expression level of FLOT1 protein is strongly correlated with the aggressive characteristic of HCC. Furthermore, patients with higher FLOT1 expression had poor overall survival and relapse-free survival. Taken together, these data not only suggested that FLOT1 can be used as a marker to identify subsets of HCC patients with more aggressive disease, but also indicated that FLOT1 might play an important role in the progression and invasion of HCC. Furthermore, our observations also provide new insight for understanding dynamic balance of lipid rafts instability in HCC and highlight the important role of flotillin proteins in the development and progression of HCC.

The overall 5-year survival rate of HCC patients remains poor, which is largely attributable to the high rates of extensive vascular invasion or extrahepatic spread [3]. Serum α-fetoprotein (AFP), a fetal-specific glycoprotein antigen, is the most widely used diagnostic and prognosis predictive tumor marker for patients with HCC. A randomized controlled trial in China reveals that HCC surveillance with testing of serum AFP and performance of abdominal ultrasound (US) at repeated 6-month intervals significantly improves patient survival [25], [26]. However, the reported sensitivity of AFP for detecting HCC varies widely in both HBV-positive and HBV-negative populations [27]. The sensitivity and specificity of AFP varies from 39% to 97% and 76% to 95%, respectively [28], [29]. In addition, AFP is a fairly specific but insensitive marker for HCC. Serum AFP titers also rise in acute or chronic hepatitis, pregnancy and presence of germ cell tumors [30]. The low sensitivity of AFP makes it limited in the diagnosis and prognosis of HCC [31]. Herein, we found that FLOT1 could be used as a valuable prognosis marker independent of serum AFP. Thus, testing FLOT1 may be a useful marker for formulating prognosis and guiding the follow-up schedule in HCC patients with HCC.

In conclusion, this is the first study aimed at evaluating the possibility of using FLOT1 as a clinically relevant indicator for aggressive characteristics of HCC and as a prognostic marker for patient survival in HCC. Nevertheless, further investigation on the mechanism by which FLOT1 is involved in the development and progression of HCC and prospective studies on the prognostic significance of FLOT1 are required.
